# Zirconia-Doped Methylated Silica Membranes via Sol-Gel Process: Microstructure and Hydrogen Permselectivity

**DOI:** 10.3390/nano12132159

**Published:** 2022-06-23

**Authors:** Lintao Wang, Jing Yang

**Affiliations:** School of Urban Planning and Municipal Engineering, Xi’an Polytechnic University, Xi’an 710048, China; wanglt912@163.com

**Keywords:** microporous membrane, H_2_ permselectivity, zirconia-doped SiO_2_ membrane, hydrothermal stability, regeneration

## Abstract

In order to obtain a steam-stable hydrogen permselectivity membrane, with tetraethylorthosilicate (TEOS) as the silicon source, zirconium nitrate pentahydrate (Zr(NO_3_)_4_·5H_2_O) as the zirconium source, and methyltriethoxysilane (MTES) as the hydrophobic modifier, the methyl-modified ZrO_2_-SiO_2_ (ZrO_2_-MSiO_2_) membranes were prepared via the sol-gel method. The microstructure and gas permeance of the ZrO_2_-MSiO_2_ membranes were studied. The physical-chemical properties of the membranes were characterized by Fourier transform infrared spectroscopy (FTIR), X-ray photoelectron spectroscopy (XPS), X-ray diffraction (XRD), transmission electron microscopy (TEM), scanning electron microscope (SEM), and N_2_ adsorption–desorption analysis. The hydrogen permselectivity of ZrO_2_-MSiO_2_ membranes was evaluated with Zr content, temperature, pressure difference, drying control chemical additive (glycerol) content, and hydrothermal stability as the inferred factors. XRD and pore structure analysis revealed that, as n_Zr_ increased, the MSiO_2_ peak gradually shifted to a higher 2θ value, and the intensity gradually decreased. The study found that the permeation mechanism of H_2_ and other gases is mainly based on the activation–diffusion mechanism. The separation of H_2_ is facilitated by an increase in temperature. The ZrO_2_-MSiO_2_ membrane with n_Zr_ = 0.15 has a better pore structure and a suitable ratio of micropores to mesopores, which improved the gas permselectivities. At 200 °C, the H_2_ permeance of MSiO_2_ and ZrO_2_-MSiO_2_ membranes was 3.66 × 10^−6^ and 6.46 × 10^−6^ mol·m^−2^·s^−1^·Pa^−1^, respectively. Compared with the MSiO_2_ membrane, the H_2_/CO_2_ and H_2_/N_2_ permselectivities of the ZrO_2_-MSiO_2_ membrane were improved by 79.18% and 26.75%, respectively. The added amount of glycerol as the drying control chemical additive increased from 20% to 30%, the permeance of H_2_ decreased by 11.55%, and the permselectivities of H_2_/CO_2_ and H_2_/N_2_ rose by 2.14% and 0.28%, respectively. The final results demonstrate that the ZrO_2_-MSiO_2_ membrane possesses excellent hydrothermal stability and regeneration capability.

## 1. Introduction

It is well-known that hydrogen is a clean energy source [1]. At present, there are many ways to obtain H_2_, but the biggest problem preventing its commercialization is the purification and separation of H_2_. The purification of H_2_ can be achieved in three main ways: pressure swing adsorption, cryogenic distillation, and membrane separation [2,3]. Although pressure swing adsorption and cryogenic distillation can be operated commercially, the economic benefits are low. The main commercial application of membranes in gas separation is the separation of hydrogen from nitrogen, methane, and argon in an ammonia sweep gas stream. In the past few years, hundreds of new polymer materials have been reported, and only eight or nine polymer materials have been used to make gas separation membrane bases. Surprisingly few of them were used to make industrial membranes [4]. Membrane separation technology is also one of the most promising hydrogen purification methods. At high temperatures, the H_2_ separation membrane has attracted much attention in the application of membrane reactors, for example, in the steam reforming of natural gas. The characteristics of lower energy consumption and investment cost, as well as simple operation, have made the membrane separation method widely concerned. Compared to other techniques of hydrogen purification, the membrane separation method offers more energy efficiency and environmental friendliness. The quality of the separation membrane directly affects the separation performance. Therefore, it is very necessary to choose suitable materials to prepare efficient and stable membrane materials [5].

In recent years, research on hydrogen separation membranes has mainly focused on molecular sieves, alloys, and microporous silica [6,7,8,9]. Prominently, silica membranes have received extensive attention for H_2_ separation due to their advantages such as high permselectivity and considerable thermal stability [10]. Amorphous silica membranes derived from sol-gel and chemical vapor deposition (CVD) methods received an enormous amount of attention [11,12]. They have a stable chemical structure and molecular sieve mechanism, which can separate hydrogen across a broad temperature range [13]. However, silica membranes have demonstrated poor steam and thermal stability. Due to the hydrophilic nature of silica membranes, if they are frequently exposed to humid, low-temperature atmospheres, the flux and permselectivity of H_2_ will largely be reduced [14,15]. The study found that the addition of hydrophobic groups can reduce the affinity of silica for water and improve gas permselectivity [15]. At this stage, methyl, vinyl, perfluorodecalin, etc., are often used in the hydrophobic modification of gas permeation separation in silica membrane, and the effect of increasing the hydrothermal stability is obvious [16]. Wei et al. [17] prepared perfluorodecyl hydrophobically modified silica membranes. The results demonstrated that the addition of perfluorodecyl made the modified silica membrane change from hydrophilic to hydrophobic. The membrane exhibited excellent hydrothermal stability at 250 °C and a water vapor molar ratio of 5%. Debarati et al. [18] hydrophobically modified the surface of ceramic membranes with polydimethylsiloxane to achieve a contact angle of 141°. It shows that the surface of the ceramic membrane with the methyl group is highly hydrophobic. Somayeh et al. [19] also demonstrated that vinyl-modified silica particles can improve the hydrophobic properties of the membrane.

It is well-known that the addition of metal oxides (such as TiO_2_ [20,21], Al_2_O_3_ [22,23], Fe_2_O_3_ [24], CoO [25], and ZrO_2_ [26,27]) can not only enhance the hydrothermal stability of the membrane, but also further improve the antifouling ability and performance of the membrane. This indicates that the incorporation of metal oxides can form mixed oxide network structures that are more stable than amorphous silica materials [28,29]. In particular, ZrO_2_ is an excellent transition metal oxide, which is often utilized in studies of gas membrane separation. Li et al. [30] prepared a zirconia membrane via the polymeric sol-gel method, which possessed H_2_ permeance of about 5 × 10^−8^ mol·m^−2^·s^−1^·Pa^−1^, H_2_/CO_2_ permselectivity of 14, and outstanding hydrothermal stability under a steam pressure of 100 KPa. Gu et al. [31] used the sol-gel method to prepared the microporous zirconia membrane. After being treated with 0.50 mol·L^−1^ of H_2_SO_4_, the hydrogen permeance was (2.8 to 3.0) × 10^−6^ mol·Pa^−1^·m^−2^·s^−1^. The H_2_ permselectivities of the equimolar binary system were 6 and 9, respectively. Doping ZrO_2_ in the SiO_2_ matrix can improved the hydrophobicity and hydrothermal stability of the membrane matrix, and further enhance the gas permeability. Numerous studies have been carried out on ZrO_2_-doped silica materials/membranes. According to mesoporous stabilized zirconia intermediate layers, Gestel et al. [32] revealed a much better membrane setup. For various CO_2_/N_2_ combinations, the as-prepared membrane demonstrated permselectivities of 20–30 and CO_2_ permeances of 1.5 to 4 m^3^/(m^2^∙h∙bar). Ahn et al. [33] prepared a silica-zirconia membrane with hydrogen permselectivity on a porous alumina support using tetraethyl orthosilicate (TEOS) and tert-butanol zirconium (IV) under 923 K conditions by chemical vapor deposition. The H_2_ permeance of the obtained membrane was 3.8 × 10^−7^ mol·m^−2^·s^−1^·Pa^−1^, and the permselectivities for CO_2_ and N_2_ were 1100 and 1400, respectively. Hove et al. [34] compared the gas permeation properties of a hybrid silica (BTESE) membrane, Zr-doped BTESE membrane, and silica membrane before and after hydrothermal treatment under the same circumstances. At 100 °C, the hybrid silica membrane and Zr-doped BTESE membrane maintained good hydrothermal stability, while the silica membrane lost selectivity for all the studied gases. After hydrothermal treatment at 200 or 300 °C, the CO_2_ permeance of the Zr-doped BTESE membrane decreased significantly, and the H_2_/CO_2_ permselectivity increased significantly, by 65.71%. So far, many scholars have demonstrated the effect of different conditions during preparation on the properties of zirconia-doped silica materials/membranes. The influence of the Zr/Si molar ratio on the microstructure of the membrane and the permeability of the gas is crucial. Unfortunately, there are few reports in this regard. Furthermore, the effects of methyl modification on the microstructure and steam stability of ZrO_2_-SiO_2_ membranes were rarely described in papers. Some scholars have found that adding a drying control chemical additive (DCCA) in the process of preparing the membrane via the sol-gel method can effectively reduce the uneven shrinkage of the membrane during the heating process and during the calcining process [35], and improve the gas permselectivity of the membrane.

In this paper, methyl-modified ZrO_2_-SiO_2_ (ZrO_2_-MSiO_2_) materials/membranes with various Zr/Si molar ratios (n_Zr_) were fabricated. Glycerol was chosen to be the DCCA. The impact of n_Zr_ on the microstructures and H_2_ permselectivities of ZrO_2_-MSiO_2_ membranes was thoroughly addressed. The water vapor stability of ZrO_2_-MSiO_2_ membranes was investigated further by comparing the gas permeability characteristics of the ZrO_2_-MSiO_2_ membranes before and after steam treatment. The heat regeneration performance of ZrO_2_-MSiO_2_ membranes was also investigated.

## 2. Materials and Methods

### 2.1. Preparation of MSiO_2_ Sols

The MSiO_2_ sols were prepared by tetraethylorthosilicate (TEOS, purchased from Xi’an chemical reagent Co., Ltd., Xi’an, China) as a silica source, methyltriethoxysilane (MTES, purchased from Hangzhou Guibao Chemical Co., Ltd., Hangzhou, China) as a hydrophobic modified agent, anhydrous ethanol (EtOH, purchased from Tianjin Branch Micro-Europe Chemical Reagent Co., Ltd., Tianjin, China) as a solvent, and nitric acid (HNO_3_, purchased from Sichuan Xilong Reagent Co., Ltd., Chengdu, China) as a catalyst. To begin, TEOS, MTES, and EtOH were completely combined in a three-necked flask using a magnetic stirrer. The flask was correctly immersed in an ice-water combination. The solution was then agitated for 50 min using a magnetic stirrer to ensure thorough mixing. The H_2_O and HNO_3_ combination was then dropped into the mixture while it was still being stirred. The reaction mixture was then agitated in a three-necked flask at a constant temperature of 60 °C for 3 h to yield the MSiO_2_ sol.

### 2.2. Preparation of ZrO_2_ Sols

In a three-necked flask, 0.6 M zirconium nitrate pentahydrate (Zr(NO_3_)_4_·5H_2_O, purchased from Tianjin Fuchen Chemical Reagent Co., Ltd., Tianjin, China) and 0.2 M oxalic acid (C_2_H_2_O_4_·2H_2_O, purchased from Tianjin HedongHongyan Chemical Reagent Co., Ltd., Tianjin, China) solutions were combined at a molar ratio of 4.5:1.0 and agitated. The aforementioned mixture was then treated with 35% (*v*/*v*) glycerol (GL, purchased from Tianjin Kemiou Chemical Reagent Co., Ltd., Tianjin, China), and stirring was maintained in a water bath at 50 °C for 3 h to yield the ZrO_2_ sols.

### 2.3. Preparation of ZrO_2_-MSiO_2_ Sols

The ZrO_2_ sols were aged for 12 h at 25 °C. The ZrO_2_ sols and EtOH were then added to the MSiO_2_ sols and stirred for 60 min to create the required ZrO_2_-MSiO_2_ sols. The n_Zr_ ratio was 0, 0.08, 0.15, 0.3, and 0.05. The ZrO_2_-MSiO_2_ sols were diluted three times with ethanol after 12 h. GL was used as a drying control agent at 0%, 10%, 20%, and 30% (DCCA). After 60 min of stirring, ZrO_2_-MSiO_2_ sols with varied GL contents were obtained.

### 2.4. Preparation of ZrO_2_-MSiO_2_ Materials

The ZrO_2_-MSiO_2_ sols were then placed individually in petri plates for gelation at 30 °C. The gel materials were ground and pulverized with a mortar, and then calcined at a heating rate of 0.5 °C·min^−1^ at 400 °C for 2 h under nitrogen atmosphere protection, and then cooled down naturally. The ZrO_2_-MSiO_2_ materials with different n_Zr_ were prepared. The ZrO_2_-MSiO_2_ materials with n_Zr_ = 0 are also referred to as “MSiO_2_” materials.

### 2.5. Preparation of ZrO_2_-MSiO_2_ Membranes

**Figure 1:**
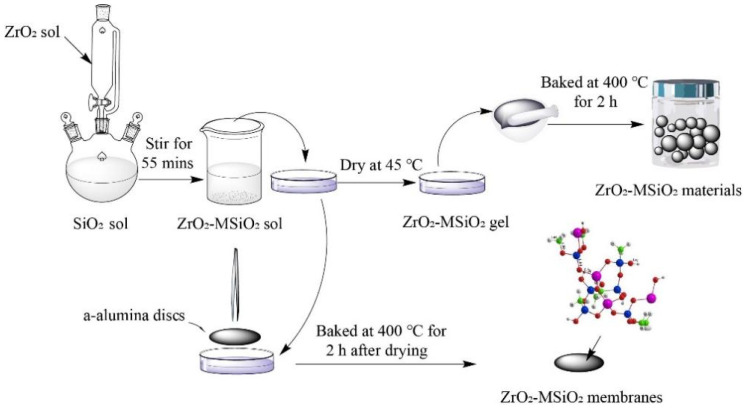
Schematic diagram of the preparation of ZrO_2_-MSiO_2_ materials/membranes.

The ZrO_2_-MSiO_2_ membranes were coated on top of composite interlayers supported by porous α-alumina discs. The discs are 5 mm-thick and 30 mm in diameter, with a porosity of 40% and an average pore size of 100 nm. ZrO_2_-MSiO_2_ membranes were effectively prepared by dip-coating the substrates in three-fold ethanol-diluted silica sol for 7 s, then drying and calcining them. Each sample was dried at 30 °C for 3 h before being calcined at 400 °C in a temperature-controlled furnace in a N_2_ environment with a ramping rate of 0.5 °C·min^−1^ and a dwell period of 2 h. The dip-coating-drying-calcining process was repeated three times. Figure 1 demonstrates the preparation process of the ZrO_2_-MSiO_2_ materials/membranes. The ZrO_2_-MSiO_2_ membranes with n_Zr_ = 0 are also referred to as “MSiO_2_” membranes.

### 2.6. Steam Treatment and Regeneration of ZrO_2_-MSiO_2_ Membranes

The ZrO_2_-MSiO_2_ membranes were subjected to a 7-day steam stability test in which they were placed into saturated steam at 25 °C. After steam treatment, for thermal regeneration of ZrO_2_-MSiO_2_ membranes, they were processed at a calcination temperature of 350 °C, with the same calcination technique as before. The gas permeances of ZrO_2_-MSiO_2_ membranes were investigated after steam treatment and regeneration, respectively.

### 2.7. Characterizations

Using Fourier transform infrared spectroscopy, the functional groups of ZrO_2_-MSiO_2_ materials were characterized (FTIR, Spotlight 400 and Frontier, PerkinElmer Corporation, Waltham, MA, USA), and the wavelength measuring range was 400 to 4000 cm^−1^ using the KBr compression technique. Using a Rigaku D/max-2550pc X-ray diffractometer (XRD, Rigaku D/max-2550pc, Hitachi, Tokyo, Japan) with CuKα radiation at 40 kV and 40 mA, the ZrO_2_-MSiO_2_ materials’ phase structure was found. The X-ray photoelectron spectra (XPS) were acquired on a K-Alpha X-ray photoelectron spectroscope from Thermo Fisher Scientific with AlKα excitation and were calibrated regarding the signal of adventitious carbon (XPS, ESCALAB250xi, Thermo Scientific, Waltham, MA, USA). The binding energy estimates were derived using the C (1s) line at 284.6 eV as the reference point. Transmission electron microscopy (TEM, JEM 2100F, JEOL, Tokyo, Japan) was utilized to investigate the ZrO_2_-MSiO_2_ powders’ crystallization. Operating at 5 kV, scanning electron microscopy (SEM, JEOL JSM-6300, Hitachi, Tokyo, Japan) was utilized to study the surface morphologies of the ZrO_2_-MSiO_2_ membranes. N_2_ adsorption–desorption measurements were conducted using an automated Micromeritics, ASAP2020 analyzer (ASAP 2020, Micromeritics, Norcross, GA, USA). The ZrO_2_-MSiO_2_ materials’ BET surface area, pore volume, and pore size distribution were determined.

**Figure 2:**
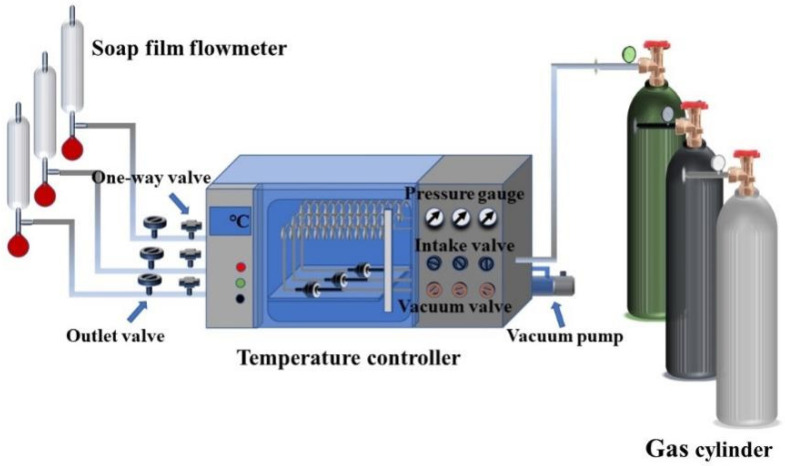
Single gas permeation experiment device diagram.

Figure 2 is a schematic of the experimental setup used to evaluate the performance of single gas permeation. Prior to the experiment, the pressure and temperature were set to the desired values for thirty minutes to allow the gas permeation to stabilize. The permeation properties of MSiO_2_ and ZrO_2_-MSiO_2_ membranes were evaluated using H_2_, CO_2_, and N_2_. The gas permeability was determined based on the outlet gas flow. The gas permselectivity values (ideal permselectivities) were calculated by the permeance ratio between two gases.

## 3. Results

### 3.1. Chemical Structure Analysis

**Figure 3:**
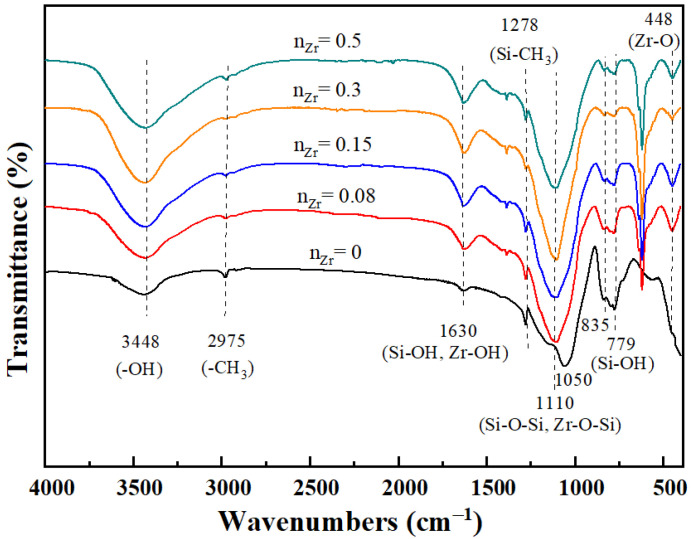
FTIR spectra curves of ZrO_2_-MSiO_2_ materials with various n_Zr_.

FTIR spectra were used to investigate the functional groups of ZrO_2_-MSiO_2_ materials. The FTIR spectra of ZrO_2_-MSiO_2_ materials containing various n_Zr_ contents are displayed in Figure 3. The absorption peak at around 3448 cm^−1^ was assigned to the stretching and bending vibration of the -OH group from the absorbed water. The absorption peak at 1630 cm^−1^ corresponds to Si-OH and Zr-OH on the surface of ZrO_2_-MSiO_2_ materials [36]. The antisymmetric stretching vibration absorption peak -CH_3_ at 2985 cm^−1^ was mainly from unhydrolyzed TEOS and MTES. The absorption peak at 1278 cm^−1^ was attributed to the Si-CH_3_ group. It is also the main hydrophobic functional group of the membrane. The absorption peak observed at 1050 cm^−1^ was attributed to the Si-O-Si bond [37]. Compared with the materials with n_Zr_ = 0, the materials with n_Zr_ = 0.08–0.5 all showed a new absorption peak at the wavenumber of 448 cm^−1^. This was related to the formation of Zr-O bonds [38]. Meanwhile, with the increase of n_Zr_, the peak at 1050 cm^−1^ shifted to around 1100 cm^−1^. This may be ascribed to the fact that partial substitution of Zr atoms for Si atoms in the Si-O-Si network to form Zr-O-Si bonds occurred [39], breaking the symmetry of SiO_2_ and leading to the shift of peak positions. However, there was no obvious Zr-O-Si bond in the FTIR spectrum of ZrO_2_-MSiO_2_ materials due to the overlap of the Zr-O-Si bond with Si-O-Si [40]. Furthermore, the decrease in the intensity of the silanol band at 779 and 835 cm^−1^ with increasing n_Zr_ could be attributed to the substitution of Si-OH bonds by Zr-O-Si bonds [41]. It demonstrates the formation of Zr-O-Si bonds in the produced materials.

### 3.2. Phase Structure Analysis

**Figure 4:**
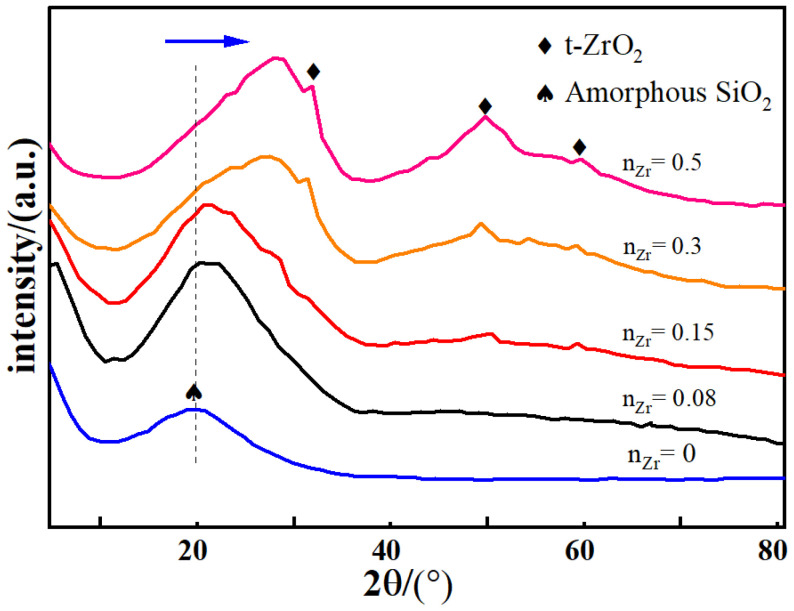
XRD patterns of ZrO_2_-MSiO_2_ materials with various n_Zr_.

The XRD patterns of the ZrO_2_-MSiO_2_ materials with varied n_Zr_ are presented in Figure 4. The peaks of amorphous SiO_2_ were concentrated at 2θ = 23.1° [42]. The SiO_2_ peak moved progressively towards higher 2θ values as n_Zr_ rose, and it slowly dropped in intensity. This is attributable to the replacement of the portion of silicon atoms by the inserted Zr atoms, producing Zr-O-Si bonds, resulting in a drop in the SiO_2_ concentration. The peaks corresponding to a crystalline tetragonal structure of zirconia are clearly apparent in the ZrO_2_-MSiO_2_ materials with n_Zr_ = 0.15–0.5. The (101), (112), and (202) reflection planes of the body-centered ZrO_2_ (t-ZrO_2_) tetragonal phase were ascribed to the large diffraction peaks occurring at 60.2°, 50.7°, and 30.2°, respectively (JCPDS No. 79-1771). XRD analysis demonstrated that with the growth of the Zr concentration, the peak intensity corresponding to t-ZrO_2_ progressively increased. In other words, the content of t-ZrO_2_ increased with the growth in Zr content. Combined with the FTIR analysis, the Zr element in ZrO_2_-MSiO_2_ materials may exist in the form of Zr-O-Si bonds and t-ZrO_2_.

**Figure 5:**
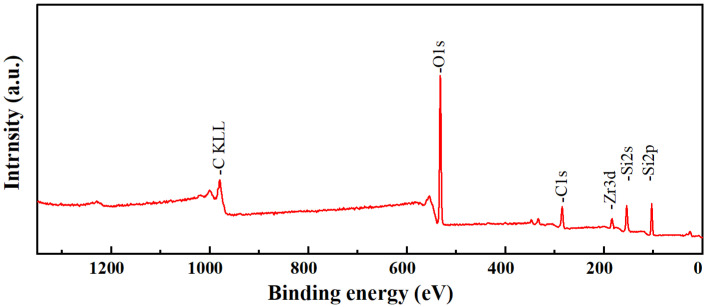
The survey XPS spectrum of ZrO_2_-MSiO_2_ materials.

**Figure 6:**
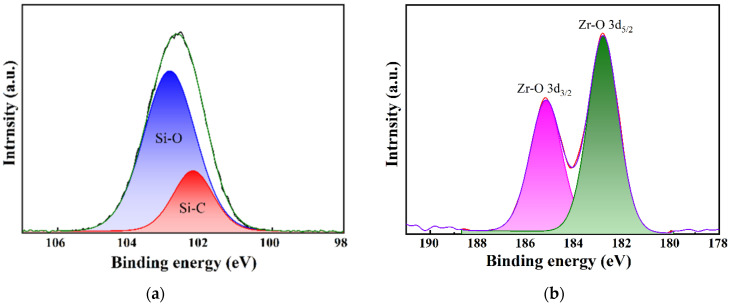
XPS peak decomposition for the (**a**) Si 2p and (**b**) Zr 3d photoelectron peaks of the ZrO_2_-MSiO_2_ materials.

To further investigate the presence of Zr and Si species in the ZrO_2_-MSiO_2_ materials, the XPS measurement was conducted. The survey XPS spectrum of ZrO_2_-MSiO_2_ material with n_Zr_ = 0.15 is shown in Figure 5. Figure 5 demonstrates that C, O, Si, and Zr elements are present in the ZrO_2_-MSiO_2_ material, which indicates the successful incorporation of Zr into the silica frameworks. Figure 6 presents the Si 2p and Zr 3d XPS spectra of the ZrO_2_-MSiO_2_ sample with n_Zr_ = 0.15. In Figure 6a, the peaks at the binding energies of 102.8 and 104.7 eV correspond to Si-C and Si-O bonds, respectively. In Figure 6b, the peaks at 186.6 and 183.3 eV correspond to the Zr-O 3d_3/2_ and Zr-O 3d_5/2_ peaks, respectively.

### 3.3. TEM Analysis

**Figure 7:**
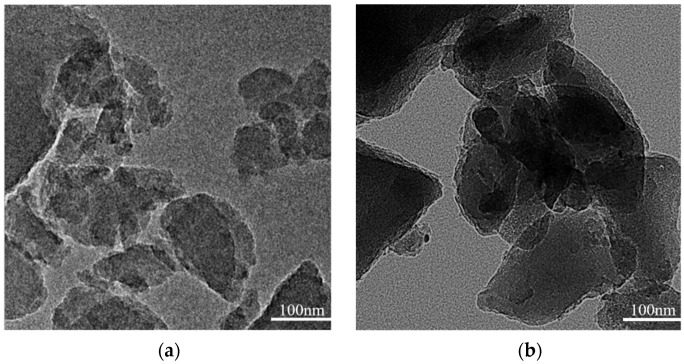
TEM images of ZrO_2_-MSiO_2_ materials with n_Zr_ = (**a**) 0 and (**b**) 0.15.

The TEM micrographs of the ZrO_2_-MSiO_2_ material with n_Zr_ = 0 and 0.15 at 400 °C under nitrogen atmosphere are illustrated in Figure 7. Figure 7a depicts that the silica particles in MSiO_2_ materials are amorphous, while in Figure 7b, a small amount of particles with darker color appear and are mixed in the silica skeleton, which may be due to the presence of t-ZrO_2_. Overall, the ZrO_2_-MSiO_2_ materials with n_Zr_ = 0.15 still maintained the amorphous state.

### 3.4. Pore Structure Analysis

**Figure 8:**
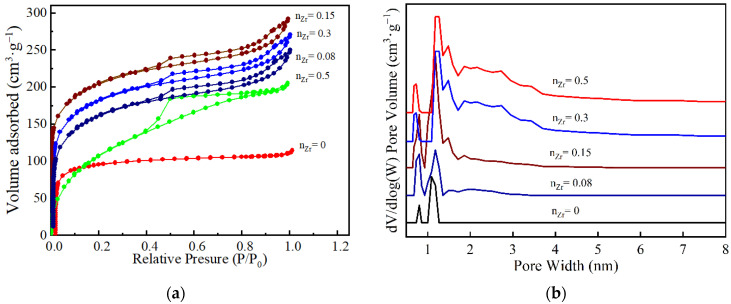
(**a**) The nitrogen adsorption-desorption isotherms and (**b**) the corresponding pore size distribution curves for the ZrO_2_-MSiO_2_ materials with various n_Zr_.

**Figure 9:**
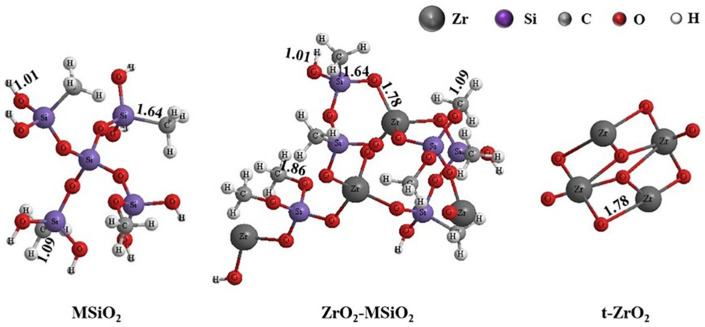
Molecular structural models of MSiO_2_, ZrO_2_-MSiO_2_, and t-ZrO_2_.

The N_2_ adsorption-desorption isotherm of the ZrO_2_-MSiO_2_ materials with various n_Zr_ are shown in Figure 8a. According to the Brunauer-Deming-Deming-Teller (BDDT) classification, the ZrO_2_-MSiO_2_ materials showed a type I adsorption isotherm, while n_Zr_ = 0 indicated the formation of microporous structures. The isotherms for the four samples (n_Zr_ = 0.08–0.5) all showed a similar trend, which could be categorized as type IV isotherms. However, the shapes of the hysteresis loops for the four samples were different, implying the variation of pore structures. A significant proportion of adsorption occurred in the range of low relative pressure, *P*/*P*_0_ < 0.1, indicating that the materials contain a large quantity of micropores. The shape of the hysteresis loop of the ZrO_2_-MSiO_2_ materials with n_Zr_ = 0.5 was altered, indicating the presence of larger mesopores or macropores. In addition, the distributions of pore size for all samples are depicted in Figure 8b. It is found that the samples with n_Zr_ = 0.08–0.5 showed a broader pore size distribution and a larger mean pore size than the samples with n_Zr_ = 0. The conclusion was also confirmed by the pore structure parameters of ZrO_2_-MSiO_2_ materials with various n_Zr_ in Table 1. The average pore size, BET specific surface area, and total pore volume of the ZrO_2_-MSiO_2_ materials gradually increased with n_Zr_ = 0.08 and 0.15, and the pore size distribution became wider. However, the total pore volume of the ZrO_2_-MSiO_2_ materials with n_Zr_ = 0.3 and 0.5 decreased instead. The fact is that the bond length of Zr-O (1.78 Å) was slightly longer than that of Si-O (1.64 Å) [43]. Figure 9 shows the molecular structure models of MSiO_2_, ZrO_2_-MSiO_2_, and t-ZrO_2_ crystallites, respectively. Hence, the formation of Zr-O-Si bonds contributes to the formation of pores. With the increase of Zr content, more and more t-ZrO_2_ crystallites were formed and distributed in the framework of MSiO_2_ materials, and the internal pore structure of the ZrO_2_-MSiO_2_ materials was hindered from shrinking and pore collapse, resulting in the decrease of the BET surface area and pore volume. From Table 1, it can be seen that the ZrO_2_-MSiO_2_ materials with n_Zr_ = 0.15 had the maximal total pore volume (0.43 cm^3^·g^−1^), BET surface area (616.77 m^2^·g^−1^), and the minimum mean pore size (2.19 nm).

### 3.5. Gas Permselectivity Analysis

#### 3.5.1. The Influence of n_Zr_

**Figure 10:**
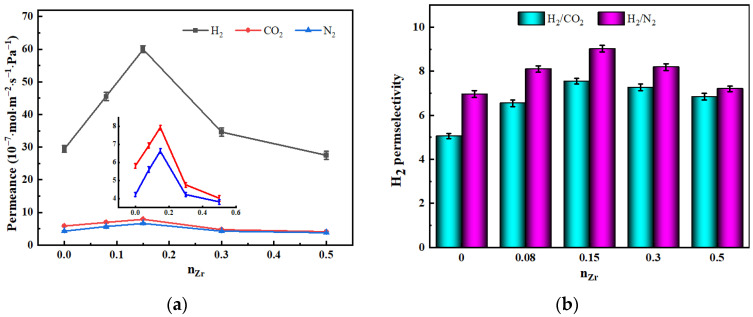
(**a**) Gas permeance and (**b**) H_2_ permselectivities of ZrO_2_-MSiO_2_ membranes with various n_Zr_ and 0% DCCA addition at a pressure difference of 0.1 MPa and 25 °C.

Figure 10 depicts the influence of n_Zr_ on the gas permeabilities and H_2_ permselectivities of ZrO_2_-MSiO_2_ membranes with varying n_Zr_ and 0% DCCA addition at 25 °C and 0.1 MPa. In Figure 10a, with the increase of n_Zr_, the H_2_, CO_2_, and N_2_ permeances of the samples increased until n_Zr_ = 0.15, and then decreased. Compared with the MSiO_2_ membranes (n_Zr_ = 0), the H_2_, CO_2_, and N_2_ permeance of the ZrO_2_-MSiO_2_ membranes with n_Zr_ = 0.15 increased by 50.95%, 26.74%, and 36.36%, respectively. From the pore structure analysis (Table 1), the overall pore volumes of the ZrO_2_-MSiO_2_ membranes grew somewhat with increasing n_Zr_ until n_Zr_ = 0.15, and then decreased, which can explain why the ZrO_2_-MSiO_2_ membranes with n_Zr_ = 0.15 had the highest permeance to each gas. For the same membrane, the order of gas molecular permeance is H_2_ > CO_2_ > N_2_. Gas permeance decreased with increasing d_k_ (0.289, 0.33, and 0.364 nm, respectively), indicating that all membranes exhibited molecular sieve properties. However, when n_Zr_ ≥ 0.15, the permeance of CO_2_ decreased more closely to that of N_2_. This behavior was related to the fact that following heat treatment at 400 °C, the Zr-O-Si bonds and t-ZrO_2_ crystallites generated in the ZrO_2_-SiO_2_ membranes will generate a significant number of Brønsted acid sites. High acidity leads to a reduction in the affinity of the membranes for CO_2_, hence lowering the CO_2_ permeance [44].

It can be observed from Figure 10b that compared with MSiO_2_ membranes, the H_2_/CO_2_ and H_2_/N_2_ permselectivities of ZrO_2_-MSiO_2_ membranes with n_Zr_ = 0.15 increased by 22.93% and 33.04%, respectively. Combined with the previous characterization test, it was found that the ZrO_2_-MSiO_2_ membranes with n_Zr_ = 0.15 had a good pore structure, which is beneficial to improve the permselectivity of gas. In addition, the acidic sites formed by the ZrO_2_-MSiO_2_ membranes reduced the affinity of the membranes for CO_2_ and helped to separate it from H_2_. However, the permselectivities of ZrO_2_-MSiO_2_ membranes after n_Zr_ = 0.15 showed a decreasing trend. Compared with the ZrO_2_-MSiO_2_ membranes with n_Zr_ = 0.15, the H_2_/CO_2_ and H_2_/N_2_ permselectivities of the membranes with n_Zr_ = 0.5 decreased by 9.35% and 20.15%, respectively. As a result, just because the n_Zr_ concentration is larger, it does not indicate that the separation effect is better. Since the Zr-O and Si-O bonds in zirconium-substituted siloxane rings are longer than in pure siloxane rings, for the ZrO_2_-MSiO_2_ membranes with n_Zr_ = 0.5, the number of siloxane rings containing Zr increased, and the pore size of the membranes became larger. Meanwhile, a large number of t-ZrO_2_ crystals were produced, which led to the shrinkage of the pore structure inside the membranes and the collapse of the pores, resulting in the decrease of the permselectivities of the membranes.

**Figure 11:**
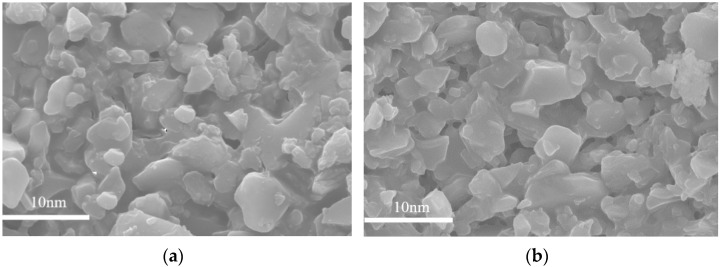
SEM images of surface topography for (**a**) MSiO_2_ and (**b**) ZrO_2_-MSiO_2_ (n_Zr_ = 0.15) membranes with 0% DCCA addition calcined at 400 °C.

SEM images of surface topography for MSiO_2_ and ZrO_2_-MSiO_2_ (n_Zr_ = 0.15) membranes calcined at 400 °C are shown in Figure 11. Compared to the MSiO_2_ membranes, the particle size and distribution of the ZrO_2_-MSiO_2_ membranes were more uniform, the membranes’ surfaces had no obvious defects, and the surface was uniform and smooth. The particle size of MSiO_2_ membranes was between 1.1 and 5.8 nm, while the particle size of ZrO_2_-MSiO_2_ membranes was between 1.3 and 8.9 nm. The formed ZrO_2_-MSiO_2_ membranes with a smooth surface and uniform membrane pores were more conducive to the gas permselectivitiy.

#### 3.5.2. The Influence of Temperature

**Figure 12:**
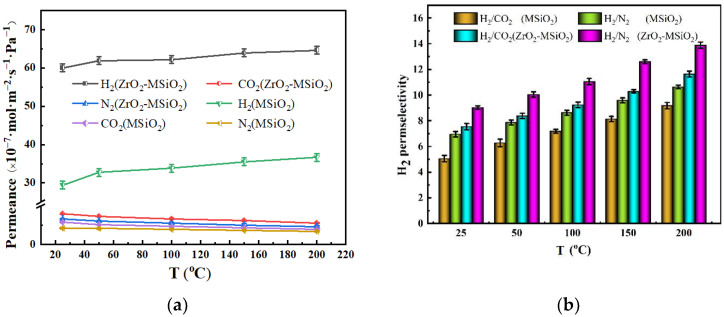
Influence of temperature on the (**a**) gas permeances and (**b**) H_2_ permselectivities of MSiO_2_ and ZrO_2_-MSiO_2_ (n_Zr_ = 0.15) membranes with 0% DCCA addition at a pressure difference of 0.1 MPa.

The permeances and permselectivities of the MSiO_2_ and ZrO_2_-MSiO_2_ (n_Zr_ = 0.15) membranes with 0% DCCA addition at a pressure difference of 0.1 MPa and temperature changing from 25 to 200 °C are shown in Figure 12. Obviously, with the increasing temperature, the permeance of H_2_ of MSiO_2_ and ZrO_2_-MSiO_2_ membranes increased gradually, as seen in Figure 12a. From 25 to 200 °C, the H_2_ permeance of MSiO_2_ and ZrO_2_-MSiO_2_ membranes rose by 19.66% and 7.12%, respectively, demonstrating that the H_2_ permeation behavior in the two membranes followed the activated diffusion transport mechanism. In contrast, the permeabilities of CO_2_ and N_2_ were similar to the Knudsen diffusion trend, whereby both slightly decreased. The CO_2_ permeance of MSiO_2_ and ZrO_2_-MSiO_2_ membranes decreased by 31.46% and 30.20% from 25 to 200 °C, respectively, and the N_2_ permeance decreased by 18.60% and 29.98%, respectively. The major explanations for the decrease in CO_2_ and N_2_ permeance were the violent movement of molecules and the rise in the mean free path as temperature increased.

In Figure 12b, it can be seen that the permselectivities in the membranes gradually increased with the increase of temperature. At 200 °C, compared with the MSiO_2_ membranes, the H_2_/CO_2_ and H_2_/N_2_ permselectivities of the ZrO_2_-MSiO_2_ membranes increased by 21.11% and 23.34%, respectively. The above results show that the ZrO_2_-MSiO_2_ membranes had better permselectivity and permeance of H_2_ than the MSiO_2_ membranes under the same conditions.

Combined with the preceding studies, it was determined that a rise in temperature facilitated the separation of H_2_ and that the separation process of H_2_ from CO_2_ and N_2_ is dominated by activation diffusion, which is described by the Arrhenius equation [45]:

(1) $$\begin{array}{l} P=P_{0}\exp(-\frac{E_{a}}{RT}) \end{array}$$

**Figure 13:**
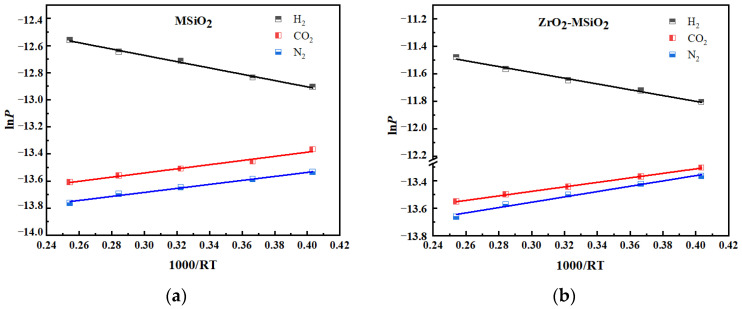
Arrhenius plots of different gases of (**a**) MSiO_2_ and (**b**) ZrO_2_-MSiO_2_ (n_Zr_ = 0.15) membranes.

In Formula (1), *P* is the permeation rate, *E*_a_ is the apparent activation energy, and *P*_0_ is a constant, which depends on the pore wall–gas molecule interaction, gas selective layer thickness, and pore shape and tortuosity [46]. For linear fitting, 1000/*RT* was used as the abscissa and ln*P* as the ordinate, and the slope of the fitting equation might be used to obtain the apparent activation energy. Figure 13 depicts the Arrhenius fitting diagrams for the three gases.

Figure 13 demonstrates that the apparent activation energy of H_2_ is positive, while that of various other gases is negative. This is related to the gas-activated transport behavior, whereby there are two parallel transmission channels for gas through the membrane: one is through selective micropores, with gas transport processed by a thermally activated surface diffusion mechanism in the micropore state [47], and the other is through larger pores [48]. The activation energies for CO_2_ and N_2_ are negative, indicating that there are permeation pathways large enough in these types of membranes to allow the diffusion of these larger gas. It is generally believed that *E*_a_ is composed of two parts [46], the adsorption heat, *Q*_st_, of gas on the surface of the membrane and the activation energy, *E*_m_, of gas flowing through the solid surface. The larger the *E*_m_ is, the harder it is for the gas to diffuse, *E*_a_ = *E*_m_ − *Q*_st_. Arrhenius equation parameter values are shown in Table 2. Table 2 shows that the *E*_m_ values of the gases in the ZrO_2_-MSiO_2_ membrane are all less than those in the MSiO_2_ membrane. It shows that the structure of ZrO_2_-MSiO_2_ membranes is not as dense as that of MSiO_2_ membranes. This is in good accordance with the N_2_ adsorption–desorption results. This finding also shows that ZrO_2_ doping successfully diminishes the densification of the SiO_2_ network. The higher porosity of the ZrO_2_-MSiO_2_ membranes allows the gas to cross the membrane pore barrier using their kinetic energy. Therefore, the gas (H_2_, CO_2_, and N_2_) permeance of ZrO_2_-MSiO_2_ membranes is higher than that of MSiO_2_ membranes.

Table 3 displays the *E*_a_ of H_2_, pore diameter, H_2_ permeance, and H_2_ permselectivities for several membranes/films prepared by other researchers. It is challenging to concurrently enhance the membranes’ permselectivity and gas permeability, as seen in Table 3. Generally, the larger the average pore size of the membrane, the higher the permeance to H_2_, which is accompanied by a smaller *E*_a_. Meanwhile, the *E*_a_ of H_2_ is related to the interaction of H_2_ with the membrane pore wall. It can be seen from Table 3 that the as-prepared ZrO_2_-MSiO_2_ membrane has a large H_2_ permselectivity compared to other membranes.

#### 3.5.3. The Influence of Pressure Difference

**Figure 14:**
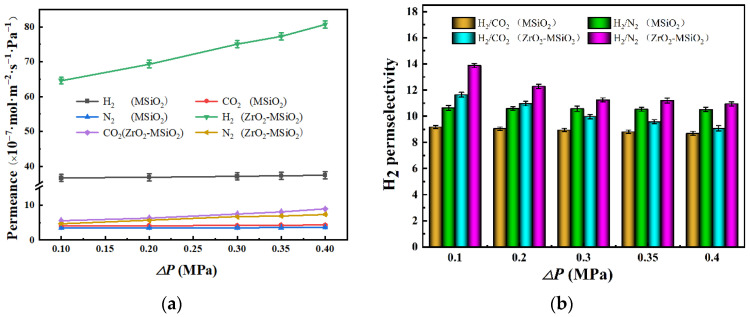
Influence of pressure difference on the (**a**) gas permeances and (**b**) H_2_ permselectivities of MSiO_2_ and ZrO_2_-MSiO_2_ (n_Zr_ = 0.15) membranes with 0% DCCA addition at 200 °C.

Figure 14 illustrates the effect of pressure difference on the gas permeances and H_2_ permselectivities of MSiO_2_ and ZrO_2_-MSiO_2_ (n_Zr_ = 0.15) membranes at 200 °C with 0% DCCA addition. In Figure 14a, it can be observed that the H_2_ permeance of the ZrO_2_-MSiO_2_ membranes with n_Zr_ = 0.15 improved with the increase of the pressure difference, and the pressure dependence increased. However, the H_2_ permeance of MSiO_2_ membranes remained basically unchanged. The MSiO_2_ and ZrO_2_-MSiO_2_ membranes increased their H_2_ permeance by 2.16% and 19.96%, respectively, when the pressure was increased from 0.10 to 0.40 MPa. In Figure 14b, the H_2_ permselectivities of MSiO_2_ membranes did not change significantly, and the H_2_/CO_2_ and H_2_/N_2_ permselectivities decreased by 5.27% and 1.12%, respectively. However, the H_2_ permselectivities of the ZrO_2_-MSiO_2_ membranes with n_Zr_ = 0.15 changed greatly, where the H_2_/CO_2_ and H_2_/N_2_ permselectivities decreased by 22.04% and 21.12%, respectively. Clearly, it can be seen that the permselectivity of the ZrO_2_-MSiO_2_ membranes was reduced more than that of the MSiO_2_ membranes, that is, the pressure had a relatively small effect on the gas permeation of the MSiO_2_ membranes. This phenomenon is attributed to the relatively dense MSiO_2_ membranes with micropores as the main component. With the increase of the pressure difference between the two sides of the membranes, the power of the gas passing through the ZrO_2_-MSiO_2_ membranes increased, making it easier for the gas to pass through the mesopores or even the macropores, which has a greater impact on the permselectivity of the membranes. At the same time, it is shown that the H_2_ diffusion mechanism of ZrO_2_-MSiO_2_ membranes was different from that of MSiO_2_ membranes due to the influence of doping ZrO_2_. The gas transport in the ZrO_2_-MSiO_2_ membranes follows the surface diffusion mechanism. In addition, when the pressure difference increased to 0.4 MPa, the permselectivities of H_2_/CO_2_ and H_2_/N_2_ were still higher than their respective Knudsen diffusion (4.69 and 3.74, respectively), indicating that they still have good gas permeation performance under high pressure.

#### 3.5.4. The Influence of the DCCA

**Figure 15:**
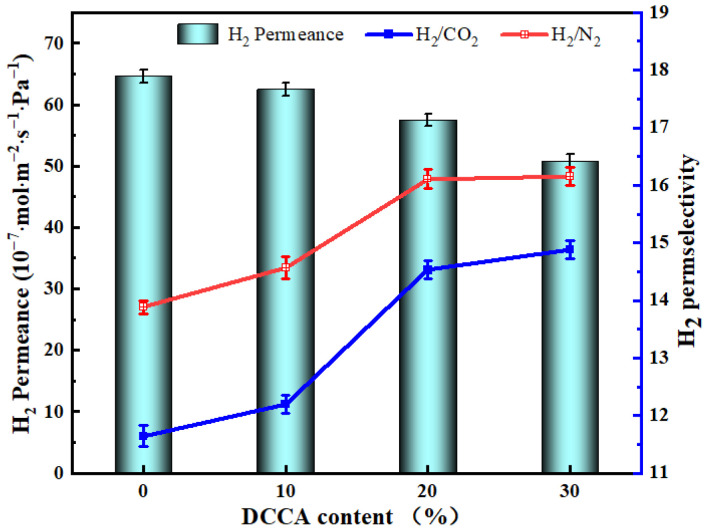
H_2_ permeances and permselectivities of ZrO_2_-MSiO_2_ membranes (n_Zr_ = 0.15) with various DCCA additions at 200 °C and 0.1 MPa.

Comparing and analyzing the previous results, it was found that the addition of DCCA (glycerol) by the sol-gel method can effectively reduce the uneven shrinkage of the membrane when it is heated during firing. The gas permeances and H_2_ permselectivities of ZrO_2_-MSiO_2_ membranes (n_Zr_ = 0.15) with various DCCA additions at 200 °C and 0.1 MPa are shown in Figure 15.

**Figure 16:**
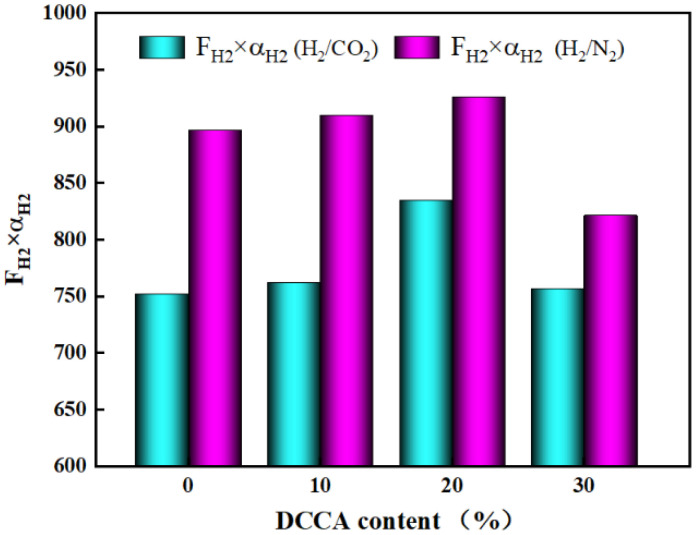
The relationship between DCCA addition and F _H2_ × α_H2_.

Figure 15 demonstrates that the H_2_ permeance of the ZrO_2_-MSiO_2_ membranes reduced by 21.30% as the DCCA addition increased from 0 to 30%. The permselectivities of H_2_/CO_2_ and H_2_/N_2_ increased by 21.77% and 14.07%, respectively. However, compared with the 20% membranes, the H_2_/CO_2_ and H_2_/N_2_ permselectivities of the membranes with the addition of 30% only increased by 2.14% and 0.28%, respectively. Figure 16 shows the relationship between DCCA (GL) contents and F_H2_ × α_H2_ (F_H2_ is the H_2_ permeance, α_H2_ is the permselective of H_2_). It was clearly observed that the addition of DCCA from 0 to 20% enhanced the F_H2_ × α_H2_ value, and after more than 20%, the F_H2_ × α_H2_ value showed a lower level. This is attributed to the fact that the addition of glycerol will gradually surround the sol particles, reduce the agglomeration caused by the collision of the colloid particles, accelerate the creation of the sol-gel network, and improve the stability of the sol structure [52]. In addition, the membrane layer collapsed and cracked easily during the drying and calcination processes, and the addition of glycerol can effectively reduce the liquid–gas surface tension to a certain extent, thereby protecting the gel skeleton from deformation [55]. However, the particles of the sol were surrounded by steric effects when too much GL was added, making the sol sticky and difficult to dry to form a membrane. The extension of the drying time makes the cross-linking of the sol more thorough and eventually leads to the densification of the membrane, which is not conducive to gas separation. From the above point of view, the membranes with 20% DCCA addition were the more worthy choice.

#### 3.5.5. Steam Treatment and Regeneration Analysis

**Figure 17:**
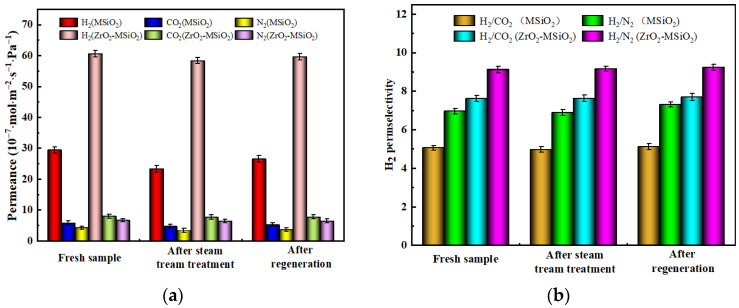
Effect of hydrothermal conditions on the (**a**) gas permeances and (**b**) H_2_ permselectivities of MSiO_2_ and ZrO_2_-MSiO_2_ (n_Zr_ = 0.15) membranes with 0% DCCA addition at a pressure difference of 0.1 MPa and 25 °C.

Figure 17 shows the effects of steam treatment and thermal regeneration on the gas permeances (H_2_, CO_2_, and N_2_) and H_2_ permselectivities of MSiO_2_ and ZrO_2_-MSiO_2_ (n_Zr_ = 0.15) membranes with 0% DCCA addition at a pressure difference of 0.1 MPa and 25 °C. The permeances of H_2_, CO_2_, and N_2_ for MSiO_2_ and ZrO_2_-MSiO_2_ membranes appear to have reduced after steam treatment. After steam aging for 7 days, the permeance of H_2_ for MSiO_2_ and ZrO_2_-MSiO_2_ membranes dropped by 20.63% and 3.70%, respectively, as compared to untreated fresh samples, and the permselectivities of H_2_/CO_2_ and H_2_/N_2_ for MSiO_2_ membranes decreased by 1.59% and 1.04%, respectively, whereas those of ZrO_2_-MSiO_2_ membranes increased by 0.09% and 0.43%, respectively.

The gas permeances (H_2_, CO_2_, and N_2_), as well as the permselectivities of H_2_/CO_2_ and H_2_/N_2_ for two membranes, all exhibit an increased trend after regeneration by calcination at 350 °C. However, as compared to untreated fresh samples, the H_2_ permeances of MSiO_2_ and ZrO_2_-MSiO_2_ membranes after regeneration dropped by 9.96% and 1.65%, respectively, whereas the permselectivities of H_2_/CO_2_ and H_2_/N_2_ for MSiO_2_ membranes improved by 1.12% and 4.71%, respectively, and those for ZrO_2_-MSiO_2_ membranes increased by 0.08% and 1.21%, respectively. The decrease in gas permeances in both membranes suggests that membrane pore shrinking occurs after calcination at 350 °C. Lower permeance and greater permselectivities are produced as a result of the smaller pores. This is attributed to the partial Zr atoms replacing the Si atoms in Si-O-Si to form more stable Zr-O-Si bonds, which further improves the hydrothermal stability of the membrane material. Therefore, the above results indicated that the ZrO_2_-MSiO_2_ membranes had a better hydrothermal stability and reproducibility than MSiO_2_ membranes.

## 4. Conclusions

The ZrO_2_-MSiO_2_ membranes were manufactured to enhance the steam stability and H_2_ permselectivity of SiO_2_ membranes. It was found that with the increase of ZrO_2_ content, the pore size distribution of the materials became wider and the average pore size increased, indicating that the doping of ZrO_2_ had the effect of expanding the pores. The ZrO_2_-MSiO_2_ membranes with n_Zr_ = 0.15 had a good pore structure and suitable micropore/mesoporous ratio, which is beneficial to improve the permeance of natural gas. At 200 °C, the H_2_/CO_2_ and H_2_/N_2_ permselectivities of ZrO_2_-MSiO_2_ membranes were 79.18% and 26.75% greater than those of MSiO_2_ membranes, respectively. Furthermore, when the pressure was increased to 0.4 MPa, the permselectivities were still higher than their respective Knudsen diffusion, indicating that they still had good gas permeance at high pressure. With the addition of DCCA from 20% to 30%, the H_2_/CO_2_ and H_2_/N_2_ permselectivities of ZrO_2_-MSiO_2_ membranes only increased by 2.14% and 0.28%, respectively, and the F_H2_ × α_H2_ value with 20% addition was the highest. In conclusion, it is worthwhile to choose 20% GL as the DCCA addition for ZrO_2_-MSiO_2_ membranes. Compared with the untreated fresh sample, after 7 days of water vapor aging, the permeance of ZrO_2_-MSiO_2_ membranes to H_2_ decreased by only 3.70%, and the permselectivities of H_2_/CO_2_ and H_2_/N_2_ increased by only 0.09% and 0.43%, respectively. After regeneration at 350 °C, the H_2_ permeance of the ZrO_2_-MSiO_2_ membranes decreased by 1.65%, and the permselectivities of H_2_/CO_2_ and H_2_/N_2_ increased by 0.08% and 1.21%. It is enough to show that the prepared ZrO_2_-MSiO_2_ membranes had a good hydrothermal stability and certain regeneration performance. In the future, the influence of high temperature (for example, ≥300 °C) and mixed gases on the gas permeances and permselectivies of ZrO_2_-MSiO_2_ membranes should be explored, which is important to the practical engineering applications.

## Figures and Tables

**Table 1:** Pore structure parameters of the ZrO_2_-MSiO_2_ materials with various n_Zr_.

n_Zr_	BET Surface Area (m^2^·g^−1^)	Average Pore Size (nm)	V_total_ (STP) (cm^3^·g^−1^)	V_micro_ (STP) (cm^3^·g^−1^)	V_micro_/V_total_ (%)
0	389.38	1.75	0.23	0.15	65.22
0.08	579.96	2.08	0.37	0.13	35.14
0.15	616.77	2.19	0.43	0.12	27.91
0.3	606.35	2.35	0.41	0.09	21.95
0.5	545.32	3.58	0.38	0.08	21.05

**Table 2:** Arrhenius equation parameter values of MSiO_2_ and ZrO_2_-MSiO_2_ (n_Zr_ = 0.15) membranes.

Membrane	Gases	*E*_a_ (kJ·mol ^−1^)	*Q*_st_ (kJ·mol^−1^)	*E*_m_ (kJ·mol^−1^)
MSiO_2_	H_2_	2.32	6.00	8.32
	CO_2_	−1.53	24.00	22.47
	N_2_	−1.48	18.00	16.52
ZrO_2_-MSiO_2_	H_2_	2.10	6.00	8.10
	CO_2_	−1.64	24.00	22.36
	N_2_	−1.94	18.00	16.06

**Table 3:** *E*_a_ of H_2_, pore diameter, H_2_ permeance, and H_2_ permselectivities for various membranes/films prepared by other researchers.

Type	Temperature/Pressure	*E*_a_ of H_2_ (kJ·mol^−1^)	Pore Diameter (nm)	H_2_ Permeance (mol·m^−2^·s^−1^·Pa^−1^)	H_2_ Permselectivities	
					H_2_/CO_2_	H_2_/N_2_
SiO_2_ [49]	200 °C, 2 bar	-	0.30–0.54	4.62 × 10^−7^	3.7	10.5
ZIF-7-SiO_2_ [50]	200 °C	-	5	8 × 10^−7^	8.78	11.8
Pd-SiO_2_ [51]	200 °C, 0.3 MPa	-	0.57	7.26 × 10^−7^	4.3	14
ZrO_2_ [52]	350 °C	-	4.95	5.3 × 10^−8^	14.3	3.1
ZrO_2_-SiO_2_ [27]	550 °C	7.0	0.165	1.8 × 10^−7^	-	-
BTDA-DDS polyimide [53]	30 °C, 5 MPa	-	-	2.52 × 10^−9^	5.16	193.21
Cellulose acetate [54]	25 °C, 1 bar	-	-	3.55 × 10^−9^	-	30.3
ZrO_2_-MSiO_2_ *	200 °C, 0.1 MPa	2.10	2.19	6.46 × 10^−6^	11.64	13.88

* In this work.

## Data Availability

Not applicable.
